# MamX encoded by the *mamXY* operon is involved in control of magnetosome maturation in *Magnetospirillum gryphiswaldense* MSR-1

**DOI:** 10.1186/1471-2180-13-203

**Published:** 2013-09-11

**Authors:** Jing Yang, Shuqi Li, Xiuliang Huang, Jinhua Li, Li Li, Yongxin Pan, Ying Li

**Affiliations:** 1 State Key Laboratories for Agro-biotechnology and College of Biological Sciences, China Agricultural University, Beijing 100193, P. R. China; 2 Institute of Geology and Geophysics, Chinese Academy of Sciences, Beijing 100029, P. R. China; 3 France-China Biomineralization and Nano-structure Laboratory, Beijing 100193, P. R.China

**Keywords:** *Magnetospirillum gryphiswaldense*, *mamXY* operon, *mamX*, Magnetosome, Crystal maturation

## Abstract

**Background:**

Magnetotactic bacteria produce membrane-enveloped magnetite crystals (magnetosomes) whose formation is controlled primarily by a gene island termed the magnetosome island (MAI). Characterization of single gene and operon function in MAI has elucidated in part the genetic basis of magnetosome formation. The *mamX* gene, located in the *mamXY* operon, is highly conserved in the MAI of all *Magnetospirillum* strains studied to date. Little is known regarding the function of *mamX* in the process of biomineralization.

**Results:**

A *mamX* deletion mutant (∆*mamX*) and its complemented strain (C*mamX*) by conjugation in *M. gryphiswaldense* strain MSR-1 were constructed. There were no striking differences in cell growth among ∆*mamX*, C*mamX*, and wild-type strain (WT). ∆*mamX* displayed a much weaker magnetic response than WT. Transmission electron microscopy revealed the presence of irregular, superparamagnetic magnetite particles in ∆*mamX*, in contrast to regular, single-domain particles in WT and C*mamX*. The phenotype of ∆*mamX* was similar to that of an *ftsZ-like* deleted mutant and *mamXY* operon deleted mutant reported previously. Quantitative real-time RT-PCR (qPCR) results indicated that the deletion of *mamX* had differential effects on the transcription levels of the other three genes in the operon.

**Conclusions:**

The MamX protein plays an important role in controlling magnetosome size, maturation, and crystal form. The four MamXY proteins appear to have redundant functions involved in magnetosome formation. Our findings provide new insights into the coordinated function of MAI genes and operons in magnetosome formation.

## Background

Magnetotactic bacteria (MTB) produce nano-sized membrane-enveloped magnetic organelles termed magnetosomes, consisting of single-domain magnetite (Fe_3_O_4_) or greigite (Fe_3_S_4_) crystals that are integrated into one to several chains depending on the species [1,2]. MTB are aquatic prokaryotes that utilize the magnetosomes to align themselves relative to magnetic fields and swim toward favorable low-oxygen, nutrient-rich environments. This behavior is called magneto-aerotaxis [1,3].

Many studies over the past several decades have focused on the molecular mechanism of magnetosome formation and revealed several important facts. Magnetosome-related genes are concentrated in a structure called the “magnetosome island” (MAI) in the genomes of MTB [4,5]. In *Magnetospirillum* strains such as *M. gryphiswaldense* MSR-1, *M. magneticum* AMB-1, and *M. magnetotacticum* MS-1, the MAI conservatively contains four common gene operons: *mms6*, *mamGFDC*, *mamAB*, and *mamXY*[2,6]. The *mamXY* operon is also conserved in *Magnetococcus* sp. MC-1 [7]. Mms6, a tightly bound protein found in the magnetosome membrane, plays an essential role in the control of magnetite crystallization and crystal size [8-10]. The MamGFDC proteins have partially redundant and collective functions in the control of magnetosome size [11]. The *mamAB* operon is a large cluster containing most of the MTB-specific genes, including those that encode the proteins MamE (involved in the localization of magnetosome membrane protein [MMP]), MamK (actin-like protein involved in the alignment of magnetosome chains), and MamJ (interacts with MamK, an important factor in magnetosome chain formation) [12-15]. Recent studies have shown that the *mamAB* operon is necessary and sufficient for magnetite biomineralization [16,17].

The *mamXY* operon received less attention than *mms6*, *mamGFDC*, and *mamAB*. *mamXY* is the last cluster in the MAI and contains four sequential genes termed *mamY*, *mamX*, *mamZ*, and *ftsZ*-*like*, identified as a polycistronic transcription unit [18]. The MamXY proteins were shown to play crucial roles in magnetite biomineralization through whole operon deletion in MSR-1 [16]. Such effect was less obvious in AMB-1 [14]. MamY was reported to constrict the magnetosome membrane in AMB-1 [19]. Deletion of FtsZ-like resulted in smaller superparamagnetic particles [18]. MamZ has been predicted (without direct evidence to date) to be an ortholog of MamH and likely a permease belonging to the major facilitator superfamily. MamX has similarities to the serine-like proteases MamE and MamS, but there have been no systematic studies of its function to date. In view of the high conservation of *mamXY* in MTB, functional studies of this operon are needed to elucidate the entire MAI and its role in the mechanism of magnetosome formation. The present study is focused on the highly conserved but hitherto uncharacterized MamX protein.

## Results

### Deletion of the *mamX* gene had no effect on cell growth

**Figure 1:**
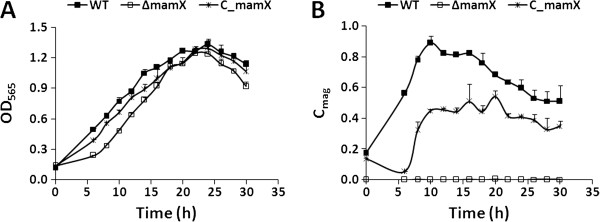
**Comparison of cell growth and magnetic response (C**_**mag**_**) in WT, mutant (∆*mamX*), and complemented strains (C*mamX*).** All experiments were performed in triplicate. **A**: There were no striking differences among the growth curves of the three strains. **B**: The C_mag_ value of ∆*mamX* was consistently zero. The C_mag_ value of WT increased from 0.17 at 0 hr to a maximum of 0.89 at 10 hr and then gradually decreased. The C_mag_ value of C*mamX* increased from 0.14 at 0 hr to 0.45 at 10 hr.

To elucidate the function of *mamX* in the absence of polar effect, MSR-1 was subjected to in-frame gene deletion (to produce strain ∆*mamX*) and complementation of *mamX* (to produce strain C*mamX*) as described in Methods. We validated the construction of the mutant and complemented strains, detected the genes in the MAI, and measured cell growth and magnetic responses. There were no notable differences in the growth curves of WT, ∆*mamX*, and C*mamX* (Figure 1A), although the OD_565_ of ∆*mamX* was slightly lower than that of WT and C*mamX* at each sample point. The maximal OD_565_ values for WT, ∆*mamX*, and C*mamX* were 1.33, 1.24, and 1.29, respectively, and were reached by 24 hr in each case.

### ∆*mamX* showed decreased intracellular iron content and magnetic response

**Figure 2:**
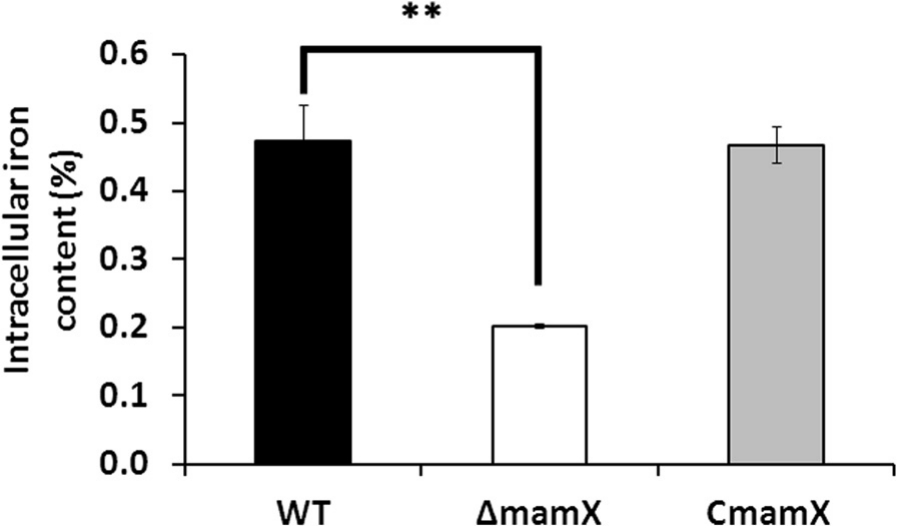
**Intracellular iron contents during culture of WT, ∆*mamX*, and C*mamX*.** The intracellular iron content was much lower for ∆*mamX* (0.20%) than for WT and C*mamX* (both 0.47%). **, The difference between WT and ∆*mamX* was statistically significant (P < 0.01, by t test).

C_mag_ can be used as an efficient value for measuring the magnetosome content of MTB [20]. For WT, C_mag_ increased from 0.17 at 0 hr to a maximum of 0.89 at 10 hr and gradually decreased thereafter (Figure 1B), while the C_mag_ value of ∆*mamX* remained zero throughout the culture period. This observation indicates a complete loss of magnetism in ∆*mamX*. C*mamX* partially recovered its C_mag_ value, which increased from 0.14 at 0 hr to 0.45 at 10 hr (Figure 1B). The complemented plasmid may exist as a free plasmid in cytoplasm rather than being integrated into the MSR-1 genome, resulting in an unstable phenotype. To further characterize the *mamX* mutant, we measured the iron content in cells. The intracellular iron content of ∆*mamX* (0.20%) was much lower than that of WT and C*mamX* (both 0.47%) (Figure 2); this difference was statistically significant (P < 0.01, by T-test).

### The deletion of *mamX* resulted in irregular and smaller crystals

**Figure 3:**
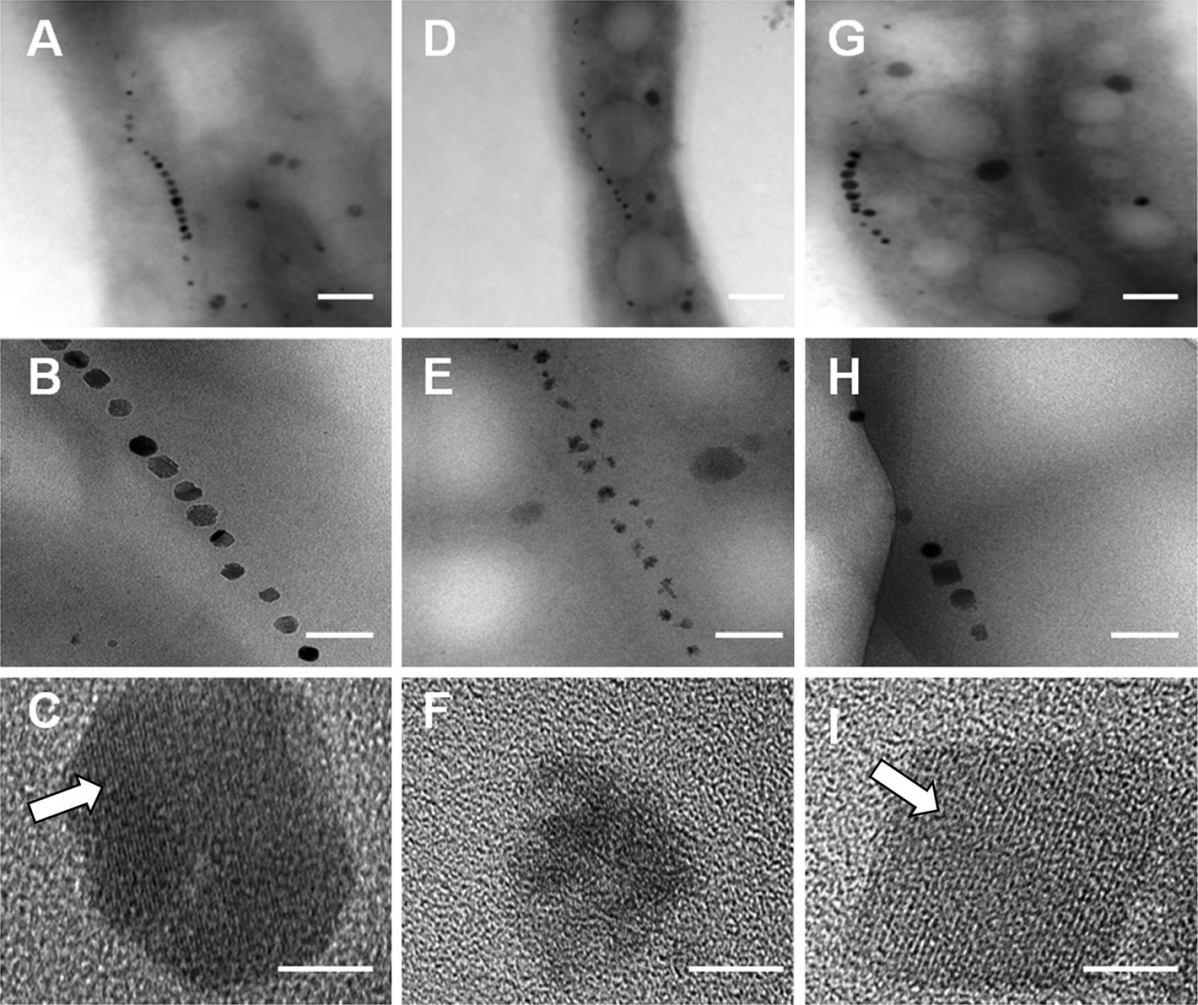
**HR-TEM observation of different cells.** HR-TEM of WT **(A, B, C)**, ∆*mamX***(D, E, F)**, and C*mamX***(G, H, I)**. **A, D, G**: cell phenotype and magnetosome location. **B, E, H**: magnetosome chain organization. **C, F, I**: crystal lattice structure. Arrows: standard Fe_3_O_4_ crystal lattice. Scale bars: **A, D, G**: 200 nm; **B, E, H**: 100 nm; **C, F, I**: 10 nm.

Phenotypic changes in the mutant cells and magnetosomes were observed by HR-TEM. WT had regular cubo-octahedral magnetosomes (mean crystal diameter 41.25±10.46 nm) (Table 1), mature chains (Figure 3A-C), and a standard magnetite crystal lattice (Figure 3C, arrow). In ∆*mamX*, the magnetosomes were much smaller (mean crystal diameter 26.11±9.92 nm) (Table 1) and irregularly shaped, and the crystal lattice was very poorly developed, although the chains were organized normally (Figure 3D-F). C*mamX* showed a normal crystal size and phenotype (mean crystal diameter 48.42±11.82 nm) (Table 1) and a typical magnetite crystal lattice (Figure 3I, arrow). The mean numbers of crystals per cell were 15.35±3.06 for WT, 20.85±3.91 for ∆*mamX*, and 6.55±1.88 for C*mamX* (Table 1). The number of intracellular magnetosomes was slightly higher in ∆*mamX* than in the other two strains. An energy-dispersive spectroscopic analysis showed that iron and oxygen were the primary elemental components of magnetosomes in ∆*mamX*, the same as in WT and C*mamX* (data not shown).

**Table 1:** Magnetosome diameters and numbers in three MSR-1 strains

**Strains**	**Maximum**	**Minimum**	**Mean**	**Mean**
	**crystal diameter**	**crystal diameter**	**crystal diameter**	**crystal number**
	**(nm)**	**(nm)**	**(nm)**	
WT	70.08	21.99	41.25 ± 10.46 ^a^	15.35 ± 3.06 ^b^
∆*mamX*	58.93	8.49	26.11 ± 9.92	20.85 ± 3.91
C*mamX*	74.91	18.14	48.42 ± 11.82	6.55 ± 1.88

For each strain, 20–30 cells and 250–300 crystals were visualized and measured. a: there is significant difference between the mean crystal diameter of WT and ∆*mamX* (P < 0.01, by Student t-test); b: there is significant difference between the mean crystal number of WT and ∆*mamX* (P < 0.01, by Student t-test).

**Figure 4:**
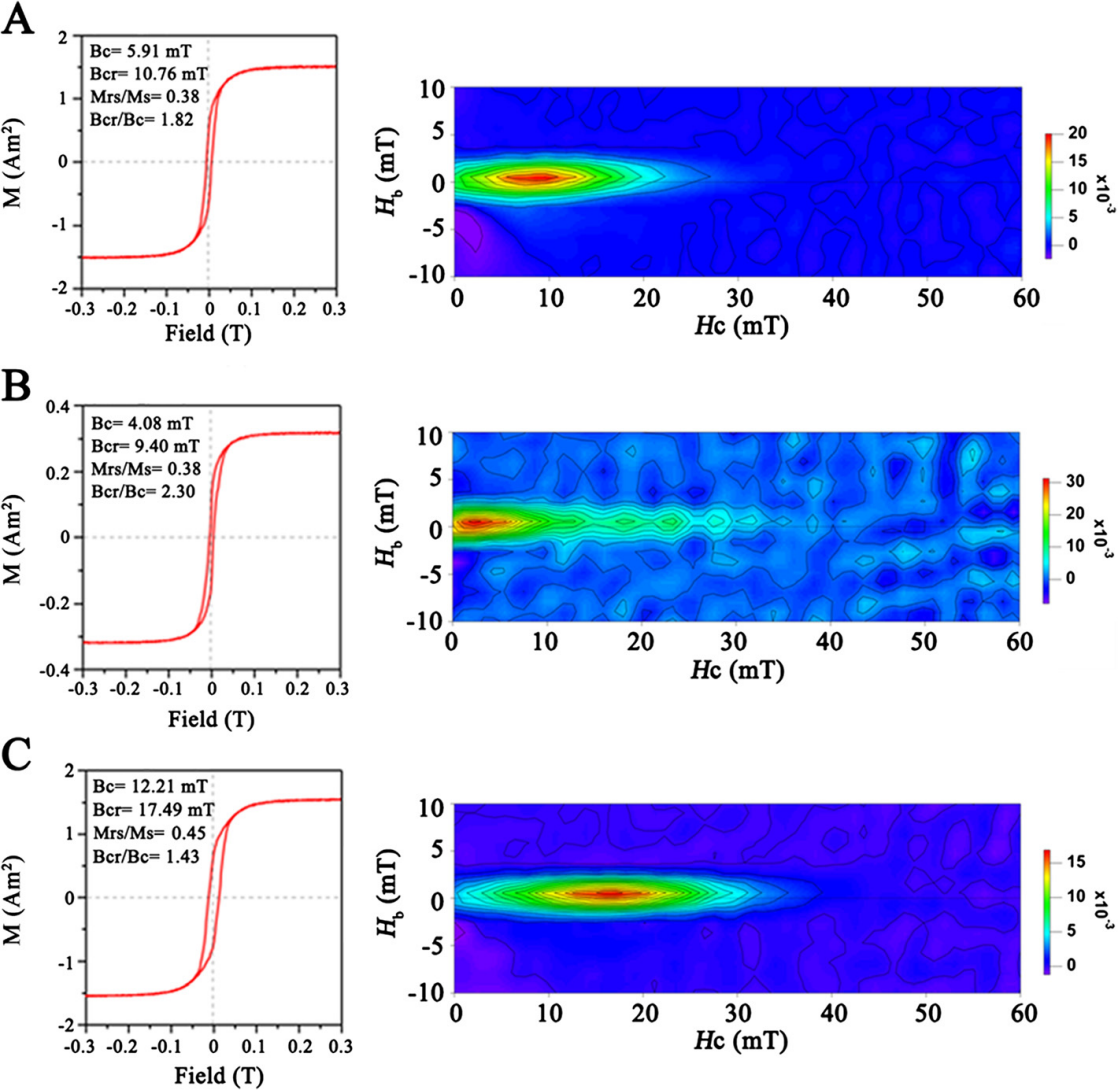
**Measurements of magnetism in deferent cells. (A)**:WT, **(B)**: ΔmamX and **(C)**: CmamX. Left: room-temperature hysteresis loops. Right: FORCs diagrams.

To further characterize the magnetosome crystals, we performed rock magnetic measurements on whole-cell samples of WT, ∆*mamX* and C*mamX* strains (Figure 4). The WT sample had a pot-bellied hysteresis loop with the hysteresis parameters coercivity *B*_c_, remanence coercivity *B*_cr_, and remanence ratio *M*_rs_/*M*_s_ being 5.91 mT, 10.76 mT, and 0.38, respectively. This indicated that the WT cell formed dominant single domain particles and small portion of superparamagnetic particles. The domain states of WT strain formed magnetosomes were further demonstrated by its corresponding first-order reversal curves (FORCs) diagram: a set of concentrated contours distributes around a peak of coercivity at *H*_c_ ≈ 9 mT and slightly intersects with the *H*_b_ axis. In contrast, the ∆*mamX* sample had a wasp-waist hysteresis loop; and its FORCs diagram slightly expanded in the horizontal distribution, but strongly intersected with the *H*_b_ axis with the peak coercivity reducing to ~2 mT. These features indicated an increased heterogeneity in microcoercivity (i.e., crystal size, morphology, and/or crystallinity) and a larger portion of superparamagnetic particles than in the WT sample [21,22]. The C*mamX* sample had Stoner-Wohlfarth-type hysteresis loop with the *M*_rs_/*M*_s_ value being 0.45; its FORC diagram was characterized by a set of closed contours concentrated around the peak coercivity of ~16 mT narrowly along the horizontal axis. These features, similar to whole-cell samples of other MTB [22-24], were typical behaviors of a randomly oriented array of non-interacting uniaxial single-domain particles [25,26]. The stronger magnetic properties (e.g., higher values of *B*_c_, *B*_cr_ and *M*_rs_/*M*_s_) exhibited by C*mamX* than WT, associated with better magnetosome formation like larger crystal size (Table 1) and/or higher crystallinity within the former than the later, was probably due to the over expression of MamX. This result, consistent with our previous study on C_*fts*Z-like strain of MSR-1 [18], further demonstrated that the *mamX* play a role in controlling the crystal size and/or crystallinity of magnetosomes within MSR-1.

### *mamXY* gene transcription levels were affected by *mamX* deletion

**Figure 5:**
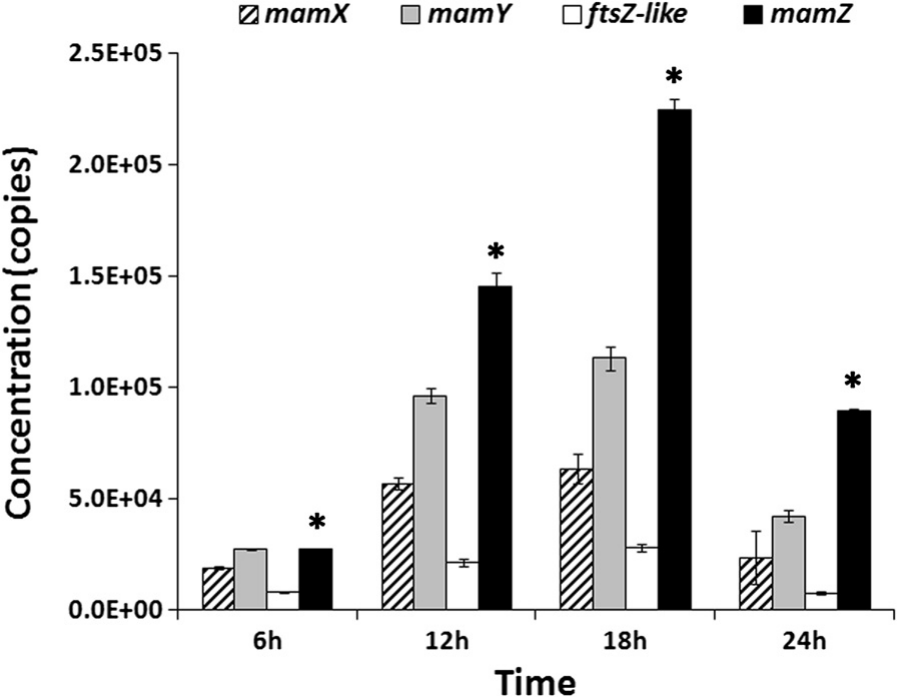
**Absolute qPCR results for transcription levels of the four genes (*mamY *, *mamX*, *mamZ*, *ftsZ-like*) in the *mamXY *operon in WT.** Each of the genes had a high transcription level from 12 to 18 hr, corresponding to the log phase of growth. The transcription level of *mamZ* was much higher than those of the other three genes at all four sampling times. *, 1/3 of original transcription level of *mamZ* in the figure was showed for better display of the other gene transcriptions.

*mamXY* gene transcription levels were evaluated in the three strains. In WT, each of the four genes (*mamY*, *mamX*, *mamZ*, and *ftsZ-like*) in the *mamXY* operon showed high transcription levels from 12 to 18 hr in absolute qPCR assay (Figure 5). This period corresponds to the log phase of growth, which is the period of rapid cell growth and magnetosome synthesis. The transcription level of *mamZ* was much higher than those of the other three genes at each of the four time points (Figure 5); *i.e.*, the level of *mamZ* was 3–6 times that of *mamY*, 4–11 times that of *mamX*, and 10–36 times that of *ftsZ-like* (Table 2). These findings suggest that the MamZ protein plays a crucial role during cell growth.

**Table 2:** **Ratio of transcription levels of MamZ to other MamXY proteins in WT and Δ*mamX *strains, based on qPCR results**

		**WT**			**Δ*mamX***	
**Ratios**		MamZ /	MamZ /	MamZ /	MamZ /	MamZ /
		MamX	MamY	FtsZ-like	MamY	FtsZ-like
	6 hr	4.0	3.0	10.0	0.4 ^a^	0.2 ^e^
	12 hr	8.0	5.0	21.0	1.6 ^b^	2.2 ^f^
	18 hr	11.0	6.0	24.0	0.8 ^c^	0.2 ^g^
	24 hr	11.0	6.0	36.0	2.9 ^d^	1.0 ^h^

a, b, c and d: ratios of taranscription level MamZ/MamY have significant differences between in WT and in Δ*mamX* strain at all the four time points (all P < 0.01, by t test); e, f, g and h: ratios of taranscription level MamZ/FtsZ-like have significant differences between in WT and in Δ*mamX* strain at all the four time points (all P < 0.01, by t test).

**Figure 6:**
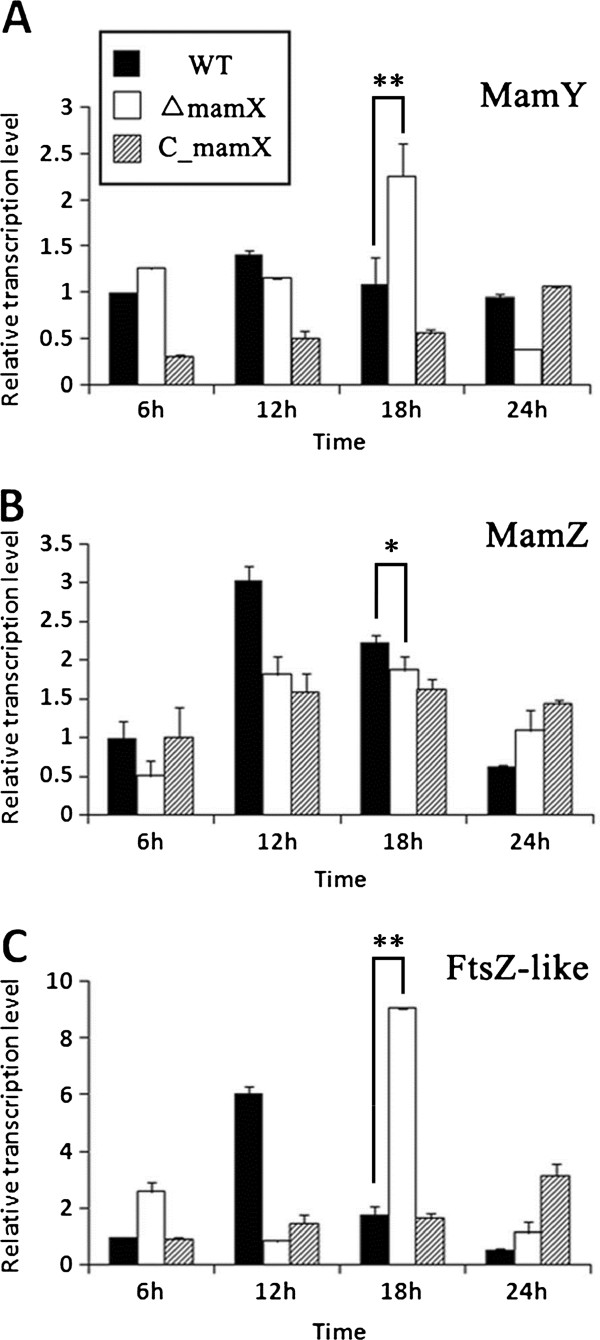
**Transcription levels of four genes in WT, Δ*mamX*, and C*mamX *strains.** All experiments were performed in triplicate. **A**: The content of MamY was similar in ∆*mamX* and WT at 6 and 12 hr but was twice as high in ∆*mamX* as in WT at 20 hr. **B**: Deletion of *mamX* had no striking effect on *mamZ* transcription. The transcriptional disparity between *mamZ* and the other three genes was large in WT but much smaller in ∆*mamX*. **C**: The level of *ftsZ*-*like* showed dramatic changes during cell growth in ∆*mamX*. The level was twice as high as in WT at 6 hr, decreased 6-fold by 12 hr, increased >4-fold by 18 hr, and then gradually declined until 24 hr. For the highest transcription of all four genes appeared at 18h in WT (see Figure 5), the Student t-test was used to analyze the differences between transcription levels of WT and ∆*mamX* at this time point. *, the difference was statistically significant (P < 0.05, by t test). **, the difference was statistically extremely significant (P < 0.01, by t test).

We used qPCR to measure the transcription levels of *mamY*, *mamZ*, and *ftsZ-like* in ∆*mamX*. The relative transcription level of *mamY* was similar in ∆*mamX* and WT at 6 and 12 hr but was twice as high in ∆*mamX* as in WT at 18 hr (Figure 6A). The transcription level of *mamZ* was much higher than those of the other three genes at all four sampling points in WT (Figure 5) but was only slightly different in ∆*mamX* (Table 2). As a result of the loss of *mamX* in the mutant, the transcription of *mamY* and *ftsZ*-like increased. The transcriptional disparity between *mamZ* and the other three genes was large in WT but much smaller in ∆*mamX* (Figure 6B; Table 2). Regardless of whether *mamX* was knocked out, the transcription level of *mamZ* was highest during the period of cell growth and high magnetosome synthesis. *ftsZ*-like showed dramatic changes of transcription level during cell growth in ∆*mamX*. Its level was twice as high as in WT at 6 hr, decreased 6-fold by 12 hr, increased >4-fold by 18 hr, and then gradually declined until 24 hr (Figure 6C). The phase of old cell division and new cell formation presumably places a high demand on the protein FtsZ-like. In summary, the deletion of *mamX* evidently resulted in higher expression of *mamY* and *ftsZ-like*, particularly at later cell growth phases, but had no major effect on the expression of *mamZ*. It should be noted that gene expression in the complemented strain C*mamX* was not identical to that in WT.

## Discussion

### MamX is involved in magnetite crystal maturation in MSR-1 cells

To elucidate the function of the highly conserved MamX protein in MTB, we constructed *mamX* deletion mutant (∆*mamX*) and complemented (C*mamX*) strains of *M. gryphiswaldense* MSR-1. For ∆*mamX*, the C_mag_ value was zero and intracellular iron content was significantly reduced, although cell growth was similar to that of WT (Figure 1). HR-TEM observations revealed that the magnetite particles in ∆*mamX* were irregularly shaped, small (26.11±9.92 nm), and predominantly superparamagnetic, whereas those in WT were symmetrically cuboid, large (41.25±10.46 nm), and predominantly single-domain. These findings indicate that MamX plays an essential role in the control of magnetosome morphology and that *mamX* is involved in magnetite crystal maturation in MSR-1.

There was a notable reduction of intracellular iron content in ∆*mamX*, corresponding to a crystal diameter much smaller than that in WT. The observed alteration of the crystal lattice may account for the reduction of C_mag_ in ∆*mamX* and result in a phenotype similar to that of a *mamXY* operon knock-out in MSR-1 [16]. Surprisingly, the mean crystal number per cell for ∆*mamX* (20.85±3.91) was 36% higher than that for WT (15.35±3.06). This finding may be due to the fact that crystals in the mutant strain were smaller; *i.e.*, equivalent amounts of materials (iron, MMP, electrons, ATP, etc.) in the cells may have been capable of producing more crystals, as supported by HR-TEM observations (Figure 3E).

### MamX has conserved double heme-binding motifs

MamX is conserved in not only spirillum strains such as *M. gryphiswaldense* MSR-1 (MGR_4149), *M. magneticum* AMB-1 (amb1017), and *M. magnetotacticum* MS-1 (MMMS1v1_36310026) but also in vibrio and cocci strains such as *Magnetovibrio* MV-1 (mv1g00028) and *Magnetococcus* sp. MC-1 (Mmc1_2238). A comparative genomic analysis showed that *mamX* is one of a set of 28 genes that are specifically associated with the magnetotactic phenotype [7]. The ubiquity and specific presence of MamX within MTB suggest that this protein plays a role in magnetotaxis. The results of the present study indicate that MamX is involved in magnetite crystal maturation but do not clarify its exact function. A protein sequence blast search using PROSITE (http://prosite.expasy.org/) showed that MamX contains two CXXCH heme-binding motifs that are typical of *c*-type cytochromes (Additional file 1: Figure S1). Similar double heme-binding motifs were found recently in the magnetosome proteins MamE, MamP, and MamT [27,28]. Site-directed mutagenesis of the two motifs in MamE resulted in the production of smaller magnetite crystals [27]. These motifs were suggested to be involved in electron transport or as a redox buffer during magnetite formation [28]. Such a function could explain the specific requirement of redox potential for magnetite formation in several MTB strains [29,30] and may be related to the function of the double heme-binding motif in MamX.

### The four proteins encoded by the *mamXY* operon may have a close relationship

The qPCR results showed that the four genes in the *mamXY* operon were all highly expressed during the log phase of growth, supporting previous findings that the log phase is an essential period for MMP function and magnetosome synthesis [31]. The expression of *mamZ* was much higher than that of the other three genes at each of the sampling times (Figure 5; Table 2), indicating that *mamZ* plays a crucial role during growth. MamZ is a highly hydrophobic protein with a predicted weight of 71.7 kDa and contains a major facilitator superfamily domain (predicted by PROSITE), a ferric reductase-like transmembrane component (Pfam; http://pfam.janelia.org/search), and up to 17 transmembrane helices (HMMTOP; http://www.enzim.hu/hmmtop). It is therefore possible that MamZ is involved in ferric iron reduction, although there is no direct experimental evidence to date for such a function. The results of the relative qPCR assay indicated that deletion of *mamX* resulted in a notable increase in *mamY* and *ftsZ-like* transcription but had no effect on *mamZ* transcription. These findings suggest some redundancy among the functions of *mamX*, *mamY*, and *ftsz-like*.

Application of the online tool STRING (http://string-db.org) predicted interactions among the four proteins encoded by the *mamXY* operon (Additional file 2: Figure S2). According to this predicted network view, the four MamXY proteins undergo intrinsic interactions with each other and are also associated with certain proteins related to cell division (MGR-2076, MGR-3226, MGR-1090, MGR-2217) and to cell wall formation (MGR-0063, MGR-1112, MGR-1092, MGR-2078, MGRGRv1-0136, MGRGRv1-0133) through FtsZ-like. These associated proteins in strain AMB-1 have predicted functions similar to those in MSR-1(Additional file 3: Table S1). Further experiments are needed to test this model.

Interestingly, the phenotypes of a *mamX* mutant, *ftsZ*-like mutant, and *mamXY* operon deleted mutant in MSR-1 are similar in that they produce magnetosomes that are small and irregularly shaped in comparison with those of WT [16,18]. In view of the previous finding that MamGFDC proteins have partially redundant and collective functions in controlling magnetosome size [11], and the results of the present study, we propose that the four genes in the *mamXY* operon have redundant functions involved in the complex process of magnetosome formation. A recent study showed that a single deletion of the *mamAB* operon in MSR-1 resulted in the complete loss of magnetosome synthesis, whereas deletion of the conserved *mms6*, *mamGFDC*, and *mamXY* operons led to severe defects in the morphology, size, and organization of magnetite crystals [16]. The MamP, MamS, MamR, and MamT proteins were shown to function in the regulation of crystal number, size, and shape [14]. Magnetite biocrystallization in MTB is clearly a complex process in which many proteins are involved. It is appropriate now to consider completing the model of MMP functions and magnetosome formation that was proposed previously [14,32].

## Conclusions

The results of the present study show that the MamX protein plays an important role in controlling magnetosome size, maturation, and crystal form. Previous studies have shown that a single gene deletion in *mamXY* and knock-out of the entire operon result in very similar phenotypic characteristics. The MamXY proteins may therefore have redundant functions involved in magnetosome synthesis. These findings are important for further elucidation of the biomineralization process in MTB.

## Methods

### Bacterial strains and growth conditions

The bacterial strains and plasmids used are listed in Table 3. *Escherichia coli* strains were cultured in Luria broth (LB) at 37°C. *M. gryphiswaldense* and its mutant strains were cultured in liquid optimized flask medium (OFM) at 30°C [33]. Sterile ferric citrate was added to OFM as an iron source after autoclaving. For conjugation, *M. gryphiswaldense* was cultured on a selection medium plate [34]. The antibiotics used were as follows: for *E. coli*, 50 μg/ml chloromycetin (Cm), 20 μg/ml gentamicin (Gm), 12.5 μg/ml tetracycline (Tc); for *M. gryphiswaldense*, the same antibiotics at concentrations of 5 μg/ml. The biomass of MSR-1 cells during culture was measured in terms of OD_565_. The magnetism of cells was measured as C_mag_ value as described previously [20].

**Table 3:** Strains and plasmids used in this study

**Strains and plasmids**	**Description**	**Source or reference**
**Strains**		
*M. gryphiswaldense* MSR-1	wild-type, Nx^r^	DSM6361
*M. gryphiswaldense* MSR-1 Δ*mamX*	*mamX* deficient mutant, Nx^r^ Gm^r^	present study
*M. gryphiswaldense* MSR-1 C*mamX*	complementation of Δ*mamX*, Nx^r^Gm^r^Tc^r^	present study
*E. coli* DH5α	*endA1 hsdR17* (r^-^ m^+^) *supE44 thi-1 recA1 gyrA* (NalR) *recA1* Δ (*lacZYA-argF*)*U169 deoR* [Ø80Δd*lacZ* ΔM15]	[35]
*E. coli* S17-1	*thi endA recA hsdR* with RP4-2-Tc::Mu-Km::Tn*7* integrated in chromosome, Sm^r^	[36]
**Plasmids**		
pUCGm	pUC1918 carrying the *aacC1* gene, Gm^r^	[37]
pSUP202	suicide vector for *M. gryphiswaldense* MSR-1, Cm^r^Tc^r^ Amp^r^	[38]
pSUPpX2	pSUP202 derivative for *mamX* deletion, Gm^r^Cm^r^Amp^r^	present study
pRK415	Cloning vector, pRK290 derivative, Tc^r^	[39]
pRK415X	pRK415 derivative for *mamX* expression, Tc^r^	present study

### Construction of the *mamX* deletion mutant and complemented strains

The *mamX* deletion mutant was constructed by conjugation and subsequent homologous recombination in MSR-1. (i) The 5′ flank (1003 bp; primers: mamX-5F, CGCGGATCCAT GTTGATGAACTTTGTCAA; mamX-5R,CGAGCTCGGGAGTTCGACTGTGGTCAA3) and 3′ flank (1043 bp; primers: mamX-3F, CGAGCTCGTGCCCTGCGTGACGACCAT; mamX-3R, ACGCGTCGACAACATTCCGAGCCAGATATA) of the *mamX* gene in the MSR-1 genome were amplified by PCR (restriction sites are underlined). The *aacC1* gene that confers Gm resistance (Gm^r^) was digested from plasmid pUCGm by *Sac*I sites. (ii) The digested and purified 5′ flank, Gm^r^, and 3′ flank were cloned into plasmid pSUP202 by *Bam*HI, *Sac*I, and *Sal* I sites to obtain the suicide plasmid pSUPpX2. (iii) *E. coli* strain S17-1 transformed with pSUPpX2 was conjugated with MSR-1 as described previously [18]. The final Gm^r^ Cm^S^ colonies, confirmed by PCR, comprised a double-crossover recombination *mamX* deletion mutant (∆*mamX*). To complement the mutant, the *mamX* gene (primers: X-F, 5′AACTGCAGTTGACCACAGTCGAACTCCC3′; X-R, 5′CGCGGATCCTATTCCATTG GGTGGGAGCG3′) was cloned into pRK415 by *Pst*I and *Bam*HI sites, and the resulting plasmid pRK415X was transferred into *E. coli* S17-1 (restriction sites are underlined). The subsequent conjugation was performed as described above. The Gm^r^ Tc^r^ colonies, confirmed by PCR, were complemented strains (termed C*mamX*).

### Transmission electron microscopy

Cells were placed on a copper grid, washed twice with distilled water, dried, and observed by TEM (Philips Tecnai F30, Eindhoven, Netherlands). For HR-TEM (JEOL 2010, Tachikawa, Tokyo), a carbon grid was used.

### Measurement of iron content

Each strain was cultured microaerobically at 30°C in OFM. After the cultures reached stationary phase, 10-ml samples were centrifuged at 10,000 x *g* for 2 min. The pellets were washed three times with distilled water, dried to a constant weight and nitrified in 1 ml nitric acid for 3 hr as described previously [40]. Intracellular iron content was assayed using an Inductively Coupled Plasma Optical Emission Spectrometer (ICP-OES; Optima 5300DV; Perkin Elmer, Waltham, MA, USA). The iron percentage of cells was calculated as iron content divided by dry weight.

### Rock magnetic measurements

Cell cultures were centrifuged (10,000 x g) at 4°C for 5 min, and the pellets were subjected to magnetic measurements. Room-temperature hysteresis loops and first-order reversal curves (FORCs) were measured by an Alternating Gradient Force Magnetometer Model MicroMag 2900 (Princeton Measurements Corp., Princeton, NJ, USA; sensitivity 1.0×10^−11^ Am^2^) as described previously [22].

### **Quantitative real-time RT-PCR** (qPCR)

Total RNA was purified using TRIzol Reagent (Invitrogen Corp., Carlsbad, CA, USA) according to the manufacturer’s instructions. The remaining genomic DNA in RNA preparations was degraded by DNase I (Takara, Shiga, Japan). cDNA synthesis was performed using M-MLV reverse transcriptase, dNTPs, and random primers (Promega Corp., San Luis Obispo, CA, USA) according to the manufacturer’s instructions.

A LightCycler 480 Instrument II (Roche, South San Francisco, CA, USA) was used for qPCR. The LightCycler 480 SYBR Green I Master kit (Roche) was used as the manual. In a 20-μl PCR system, the template cDNA content was set below 500 ng and that of each oligo as 0.5 μM. The reaction program consisted of initial denaturation at 95°C for 10 min, followed by 40 cycles of denaturation at 95°C for 15 sec, annealing at 62°C for 5 sec, extension at 72°C for 15 sec, and fluorescence measurement at 76°C for 3 sec. The reactions were repeated three more times with template cDNA serially 10-fold diluted (1/10, 1/100, and 1/1000 concentrations) to ensure that the final cDNA concentrations were between 10^3^ and 10^6^ copies. The crossing point values (Cp) were converted to absolute copies of cDNA using standard curves. The relative expressions of the target genes were calculated by dividing the absolute number of copies of cDNA by that of the reference gene *rpoc* (which encodes RNA polymerase subunit ß') in the same batch reactions. The primer sequences for qPCR are listed in Additional file 4: Table S2.

## Abbreviations

HR-TEM: High-resolution transmission electron microscopy; MAI: Magnetosome island; MMP: Magnetosome membrane protein; MTB: Magnetotactic bacteria; qPCR: Quantitative real-time RT-PCR; WT: Wild-type; OFM: Optimized flask medium; FORCs: First-order reversal curves.

## Competing interests

The authors declare that they have no competing interests.

## Authors’ contributions

JY and YL were involved in the study design. JY, SL, and XH performed the mutant construction. JL and YP performed the magnetic measurements. JY, SL, and LL performed all the other measurements. JY, SL, and YL performed the data analysis. JY and YL wrote the draft manuscript. All of the authors read and approved the final manuscript.

## Supplementary Material

Additional file 1: Figure S1 Alignments of MamX in five MTB strains. *M. magneticum* AMB-1 (amb1017), *M. magnetotacticum* MS-1 (MMMS1v1_36310026), *M. gryphiswaldense* MSR-1 (MGR_4149), *Magnetococcus* sp. MC-1 (Mmc1_2238), and *Magnetovibrio* MV-1 (mv1g00028). Identical residues are highlighted in dark gray and less conserved residues in light gray. The two boxes indicate two conserved CXXCH heme-binding motifs that are typical of *c*-type cytochromes in MamX. Click here for file

Additional file 2: Figure S2 Predicted interactions among MamX, MamY, MamZ, FtsZ-like, and related proteins. See Discussion/ “The four proteins encoded by the *mamXY* operon …” for details. Top: *mamXY* organized as a whole operon with the same promoter. Middle: molecular weights of MamXY proteins in MSR-1. Bottom: bioinformatic prediction of interactions within and outside of MamXY of MSR-1. The network nodes are proteins (green, MamY; brown, MamX; pink, MamZ; red, FtsZ-like; white, MamXY-associated proteins). The lines between two nodes represent predicted associations between two proteins. Stronger associations are represented by thicker lines. Click here for file

Additional file 3: Table S1 Predicted proteins associated with FtsZ-like in MSR-1, and the corresponding homolog proteins in *M. magneticum* AMB-1. Click here for file

Additional file 4: Table S2 Primer sequences used for quantitative real-time RT-PCR (qPCR). Click here for file
