# Supplementary material for: MamX encoded by the mamXY operon is involved in control of magnetosome maturation in Magnetospirillum gryphiswaldense MSR-1
Source: BMC Microbiol. 2013 Sep 11;13:203. doi: 10.1186/1471-2180-13-203 (PMC3847676; doi:10.1186/1471-2180-13-203)
Supplement: Additional file 1: Figure S1 — Alignments of MamX in five MTB strains. M. magneticum AMB-1 (amb1017), M. magnetotacticum MS-1 (MMMS1v1_36310026), M. gryphiswaldense MSR-1 (MGR_4149), Magnetococcus sp. MC-1 (Mmc1_2238), and Magnetovibrio MV-1 (mv1g00028). Identical residues are highlighted in dark gray and less conserved residues in light gray. The two boxes indicate two conserved CXXCH heme-binding motifs that are typical of c-type cytochromes in MamX. [file 1471-2180-13-203-S1.docx]

**Additional file1: Figure S1**


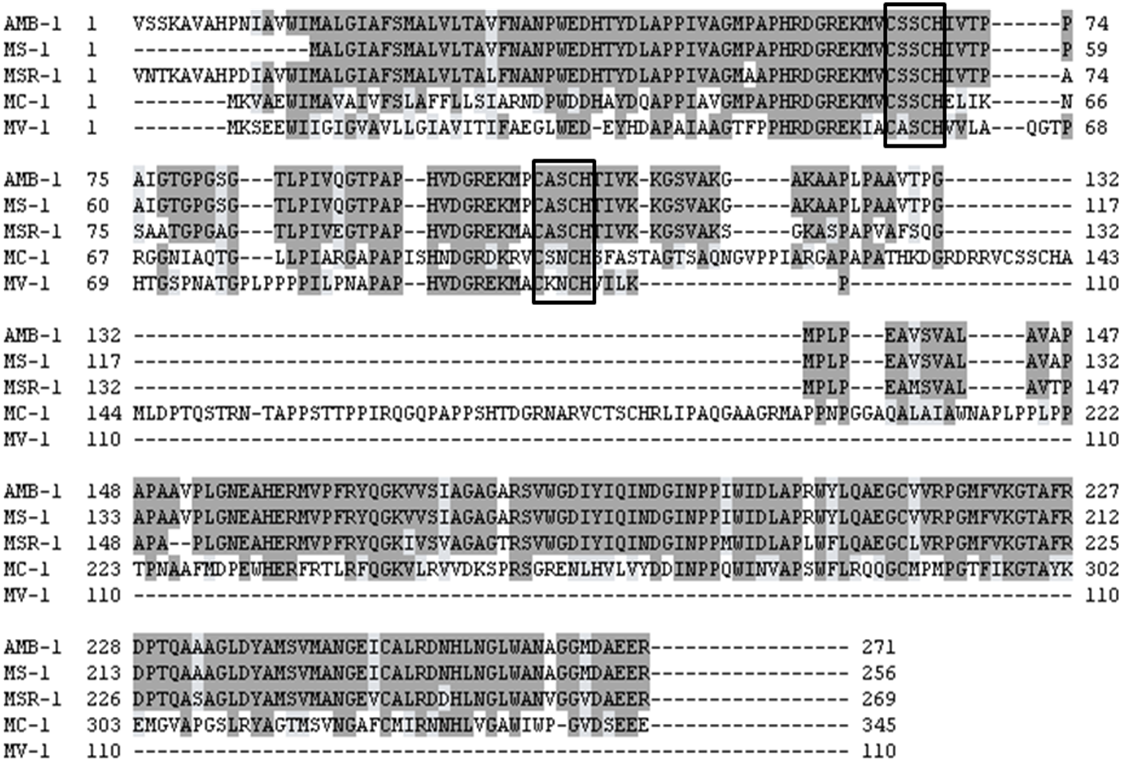


Figure S1. Alignments of MamX in five MTB strains. *M. magneticum* AMB-1 (amb1017), *M. magnetotacticum* MS-1 (MMMS1v1_36310026), *M. gryphiswaldense* MSR-1 (MGR_4149), *Magnetococcus* sp. MC-1 (Mmc1_2238), and *Magnetovibrio* MV-1 (mv1g00028). Identical residues are highlighted in dark gray and less conserved residues in light gray. The two boxes indicate two conserved CXXCH heme-binding motifs that are typical of *c*-type cytochromes in MamX.
