# Supplementary material for: MamX encoded by the mamXY operon is involved in control of magnetosome maturation in Magnetospirillum gryphiswaldense MSR-1
Source: BMC Microbiol. 2013 Sep 11;13:203. doi: 10.1186/1471-2180-13-203 (PMC3847676; doi:10.1186/1471-2180-13-203)
Supplement: Additional file 2: Figure S2 — Predicted interactions among MamX, MamY, MamZ, FtsZ-like, and related proteins. See Discussion/ “The four proteins encoded by the mamXY operon …” for details. Top: mamXY organized as a whole operon with the same promoter. Middle: molecular weights of MamXY proteins in MSR-1. Bottom: bioinformatic prediction of interactions within and outside of MamXY of MSR-1. The network nodes are proteins (green, MamY; brown, MamX; pink, MamZ; red, FtsZ-like; white, MamXY-associated proteins). The lines between two nodes represent predicted associations between two proteins. Stronger associations are represented by thicker lines. [file 1471-2180-13-203-S2.docx]

**Additional file 2: Figure S2.**


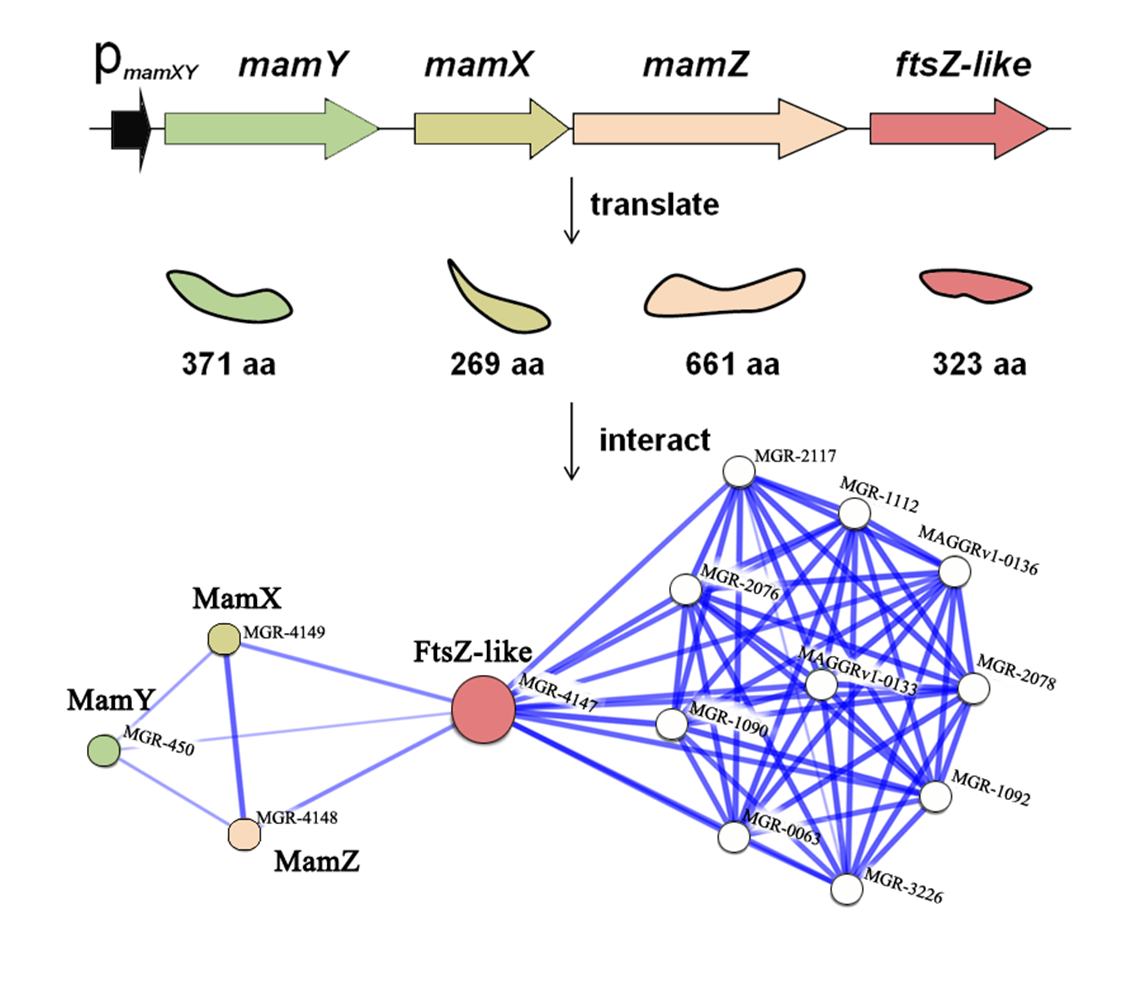
Figure S2. Predicted interactions among the four proteins and related proteins

Application of the online tool STRING (http://string-db.org) predicted interactions among the four proteins. According to this predicted network view, the MamXY proteins have intrinsic interactions with each other and are also associated with certain proteins related to cell division (MGR-2076, MGR-3226, MGR-1090, MGR-2217) and to cell wall formation (MGR-0063, MGR-1112, MGR-1092, MGR-2078, MGRGRv1-0136, MGRGRv1-0133) through FtsZ-like. These associated proteins in AMB-1 have predicted functions similar to those in MSR-1（Additional fiel 4）.

Top: *mamXY* organized as a whole operon with the same promoter. Middle: molecular weights of MamXY proteins in MSR-1. Bottom: bioinformatic prediction of interactions within and outside of MamXY of MSR-1. The network nodes are proteins (green, MamY; brown, MamX; pink, MamZ; red, FtsZ-like; white, MamXY-associated proteins). The lines between two nodes represent predicted associations between two proteins. In this predicted network view, the MamXY proteins have interactions with each other and are also associated with many proteins related to cell division and to cell wall formation (see list in Additional file 4) through FtsZ-like. Stronger associations are represented by thicker lines.
