# Supplementary material for: MamX encoded by the mamXY operon is involved in control of magnetosome maturation in Magnetospirillum gryphiswaldense MSR-1
Source: BMC Microbiol. 2013 Sep 11;13:203. doi: 10.1186/1471-2180-13-203 (PMC3847676; doi:10.1186/1471-2180-13-203)
Supplement: Additional file 3: Table S1 — Predicted proteins associated with FtsZ-like in MSR-1, and the corresponding homolog proteins in M. magneticum AMB-1. [file 1471-2180-13-203-S3.docx]

**Additional file 3: Table S1**

Table S1. Predicted proteins associated with FtsZ-like in MSR-1, and the corresponding homolog proteins in *M. magneticum* AMB-1.

|  | **In MSR-1** | | **In AMB-1** | |
| --- | --- | --- | --- | --- |
|  | **Sequence** | **Description** | **Sequence** | **Description** |
| **Cell division related proteins** | MGR-2076 | cell division protein FtsI | Amb3842 | same* |
|  | MGR-3226 | cell division protein | Amb3517 | same |
|  | MGR-1090 | cell division protein FtsA | Amb3853 | actin-like ATPase |
|  | MGR-2217 | cell cycle protein | Amb3847 | cell division protein |
| **Cell wall formation related proteins** | MGR-0063 | methyltransferase | Amb3840 | same |
|  | MGR-1112 | glycosyl transferase family 4 | Amb3845 | phospho-N- acetylmuramoyl -pentapeptide transferase |
|  | MGR-1092 | D-alanine ligase | Amb3851 | same |
|  | MGR-2078 | UDP-N-acetylmuramyl-tripeptide synthetase | Amb3843 | same |
|  | MGRGRv1-0136 | UDP-N-acetylmuramoyl-L-alanine-D-glutamate ligase | Amb3846 | same |
|  | MGRGRv1-0133 | N-acetylglucosaminyl tranferase | Amb3848 | same |

* same = same description as for MSR-1.
