# Supplementary material for: MamX encoded by the mamXY operon is involved in control of magnetosome maturation in Magnetospirillum gryphiswaldense MSR-1
Source: BMC Microbiol. 2013 Sep 11;13:203. doi: 10.1186/1471-2180-13-203 (PMC3847676; doi:10.1186/1471-2180-13-203)
Supplement: Additional file 4: Table S2 — Primer sequences used for quantitative real-time RT-PCR (qPCR). [file 1471-2180-13-203-S4.docx]

**Additional file 4 : Table S2**

Table S2.  Primer sequences used for quantitative real-time RT-PCR (qPCR).

| Primer | Sequence | Product length (bp) |
| --- | --- | --- |
| *mamY*-f | 5’- CCCAGGAAATCACCCAAGA-3’ | 246 |
| *mamY*-r | 5’- CGAAATAGGCGTTAGTCAGG-3’ |  |
| *mamZ*-f | 5’- GCCCGGTGCAGGAGATTACT-3’ | 231 |
| *mamZ*-r | 5’- TCTGGCTGTTGGTGGAGGTG-3’ |  |
| *ftsZ*-like-f | 5’- TCCGCTTGGAGGCTATGT-3’ | 236 |
| *ftsZ*-like-r | 5’- AGTGGAAGGGCTTGGTGA-3’ |  |
| *mamX*-f | 5’- CGCTGTTCAATGCCAATC-3’ | 261 |
| *mamX*-r | 5’- CAACGCTGCCCTTCTTCA-3’ |  |
| *rpoC*-f | 5’- ATCCGTATTTCCATCGCCTCCC-3’ | 160 |
| *rpoC*-r | 5’- TTGCCGCACAAGCATTCGT-3’ |  |
